# Does visual cortex lactate increase following photic stimulation in migraine without aura patients? A functional ^1^H-MRS study

**DOI:** 10.1007/s10194-011-0295-7

**Published:** 2011-02-08

**Authors:** Harmen Reyngoudt, Koen Paemeleire, Anneloor Dierickx, Benedicte Descamps, Pieter Vandemaele, Yves De Deene, Eric Achten

**Affiliations:** 1 Department of Radiology and Nuclear Medicine, MR-department (−1K12B), Ghent University Hospital, Ghent University, De Pintelaan 185, 9000 Ghent, Belgium; 2 Ghent Institute for Functional and Metabolic Imaging, Ghent University Hospital, Ghent, Belgium; 3 Department of Basic Medical Sciences, Ghent University, Ghent, Belgium; 4 Department of Neurology, Ghent University Hospital, Ghent, Belgium; 5 Laboratory for qNMR in Medicine and Biology, Ghent University, Ghent, Belgium; 6 Department of Nuclear Medicine and Radiobiology, Sherbrooke University, Sherbrooke, QC Canada

**Keywords:** Migraine without aura, Functional ^1^H-MRS, Lactate, Visual cortex, Absolute quantification

## Abstract

Proton magnetic resonance spectroscopy (^1^H-MRS) has been used in a number of studies to assess noninvasively the temporal changes of lactate (Lac) in the activated human brain. Migraine neurobiology involves lack of cortical habituation to repetitive stimuli and a mitochondrial component has been put forward. Our group has recently demonstrated a reduction in the high-energy phosphates adenosine triphosphate (ATP) and phosphocreatine (PCr) in the occipital lobe of migraine without aura (MwoA) patients, at least in a subgroup, in a phosphorus MRS (^31^P-MRS) study. In previous studies, basal Lac levels or photic stimulation (PS)-induced Lac levels were found to be increased in patients with migraine with aura (MwA) and migraine patients with visual symptoms and paraesthesia, paresia and/or dysphasia, respectively. The aim of this study was to perform functional ^1^H-MRS at 3 T in 20 MwoA patients and 20 control subjects. Repetitive visual stimulation was applied using MR-compatible goggles with 8 Hz checkerboard stimulation during 12 min. We did not observe any significant differences in signal integrals, ratios and absolute metabolite concentrations, including Lac, between MwoA patients and controls before PS. Lac also did not increase significantly during and following PS, both for MwoA patients and controls. Subtle Lac changes, smaller than the sensitivity threshold (i.e. estimated at 0.1–0.2 μmol/g at 3 T), cannot be detected by MRS. Our study does, however, argue against a significant switch to non-aerobic glucose metabolism during long-lasting PS of the visual cortex in MwoA patients.

## Introduction

Migraine is a common, disabling, primary headache disorder, with episodic manifestations that affects women three times more than men [1]. Migraine is divided into two major subtypes: migraine without aura (MwoA) and migraine with aura (MwA) [2].

The pathophysiology is still largely unknown despite a surge of interest because of the high prevalence and socio-economic impact [1]. Subcortical structures, probably including brainstem, hypothalamus and thalamus, are involved in the generation of migraine attacks [3]. Even more puzzling are the mechanisms at the basis of the interictal brain disorder that predisposes migraine patients to develop an attack. Factors such as genetic background [4–6], nitric oxide hypersensitivity [7], lack of cortical habituation [8, 9], and a disturbed energy metabolism [10] may determine the migraine threshold.

In the last 20 years, several magnetic resonance spectroscopy (MRS) studies have been performed in migraine patients. MRS is a technique to obtain in vivo biochemical information noninvasively. Many of these studies comprised phosphorus MRS (^31^P-MRS) and suggested an interictal energy disturbance in the brain of migraine patients ([10] and references herein). Other studies employed proton MRS (^1^H-MRS) and yielded a lot of heterogeneous, sometimes contradictory, results for metabolites including N-acetylaspartate (NAA: a neuronal marker), total creatine (tCr: a marker of energy metabolism), choline (Cho: a marker for membrane turnover) and myo-inositol (a glial marker) ([11] and references herein). An interesting finding was the elevated interictal level of lactate (Lac) in the occipital visual cortex of a heterogeneous group of migraine patients (i.e., MwA, basilar-type migraine, migrainous infarction) [12], suggesting a deranged oxidative glycolysis. A similar observation was made in four patients with familial hemiplegic migraine (FHM), a rare subtype of MwA [13]. In a functional ^1^H-MRS study, photic stimulation (PS) resulted in a Lac increase in the visual cortex of migraine patients with migraine with visual symptoms and paraesthesia, paresia and/or dysphasia, but not in MwA patients, in which Lac was already higher than normal in the resting state [14].

**Fig. 1:**
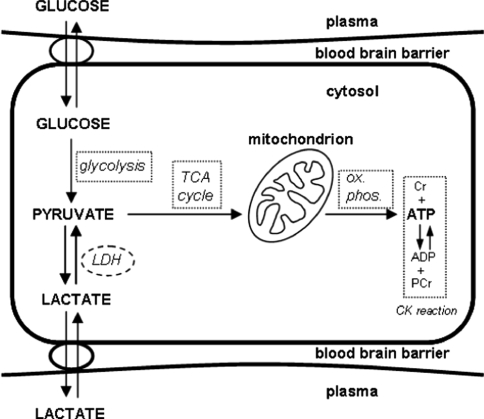
Simplified scheme of cerebral glucose metabolism. In aerobic circumstances glucose is converted into pyruvate and enters the tricarboxylic acid (*TCA*) cycle/oxidative phosphorylation, resulting in ATP production. Through the creatine kinase (*CK*) reaction, adenosine diphosphate (*ADP*) can be rephosphorylated, with conversion from phosphocreatine (*PCr*) to Cr. When aerobic energy metabolism is insufficient, there can be a switch to anaerobic glycolysis, resulting in an accumulation of Lac, enzymatically converted by Lac dehydrogenase (*LDH*)

Lac is an interesting metabolite due to its role as an intermediate of glucose metabolism and as a marker of metabolic activity under either aerobic and anaerobic conditions (Fig. 1) [15].

Therefore, the function of Lac during brain activation has been at the centre of brain energy metabolism research [16]. Fox et al. [17] found a mismatch between $\Updelta\hbox{CMRO}_2$ (variation of cerebral metabolic rate of oxygen), which increased by 5%, and $\Updelta\hbox{CMR}_{gl}$ (variation of cerebral metabolic rate of glucose) and $\Updelta$CBF (variation of cerebral blood flow), which both increased by 30–50% during prolonged neuronal activity in a positron emission tomography (PET) study. Pellerin and Magistretti [18] hypothesized that Lac, and not glucose, was the main metabolic substrate for activated neurons, as part of the so-called astrocyte-neuron Lac shuttle (ANLS). Several ^1^H-MRS studies reported an increase in Lac during prolonged PS [19–21]. However, a decrease in Lac was also observed following the presentation of an impulsive PS, partly refuting the ANLS theory [22].

Our group demonstrated a decreased level of high-energy phosphates in the occipital visual cortex of a subgroup of MwoA patients as compared to controls with ^31^P-MRS [10]. It was hypothesized that the observed adenosine triphosphate (ATP) is attributed to a reduced aerobic glycolysis and oxidative phosphorylation. This reduction in high-energy phosphates added further evidence to the hypothesis of the presence of a mitochondrial component in the pathophysiology of migraine. The question remained whether there would be an associated switch to anaerobic glycolysis, illustrated by an increase in Lac concentration. A resting state ^1^H-MRS study, however, did not show any quantifiable Lac in the visual cortex of the same MwoA patient group as in the ^31^P-MRS study [11]. The aim of this study was to investigate potential Lac changes with ^1^H-MRS in the occipital visual cortex of MwoA patients as compared to control subjects during and following PS.

## Materials and methods

### Subjects

20 MwoA patients ($31.9 \pm 9.1\,\hbox{years},$ 3 men) and 20 age- and gender-matched control subjects ($31.9 \pm 10.3\,\hbox{years},$ 3 men) were studied with functional ^1^H-MRS. All patients were recruited by the local Headache Clinic of the Ghent University Hospital where they were diagnosed with MwoA according to the criteria of the International Headache Society [2]. On average, patients experienced 3.6 ± 1.1 attacks per month, were not using any prophylactic medication and were attack-free for at least 48 h before the fMRS study. A possible attack after the study was verified by e-mail. The protocol was approved by the local ethics committee and all subjects gave written informed consent.

### ^1^H-MRS and photic stimulation

Measurements were performed on a 3 T Siemens Trio Tim whole-body scanner (Erlangen, Germany), using a 26.5-cm-diameter quadrature transmit/receive dual tuned ($^{31}P-^1H$) birdcage head coil (Rapid Biomedical, Würzburg-Rimpar, Germany). Spectra were acquired using a single voxel point-resolved spin echo sequence (PRESS), with chemical-shift-selective (CHESS) water suppression. Automatic shimming with manual fine tuning of the *B*_0_ magnetic field was used as well as iterative semi-automatic optimization of the transmitter voltage.

**Fig. 2:**
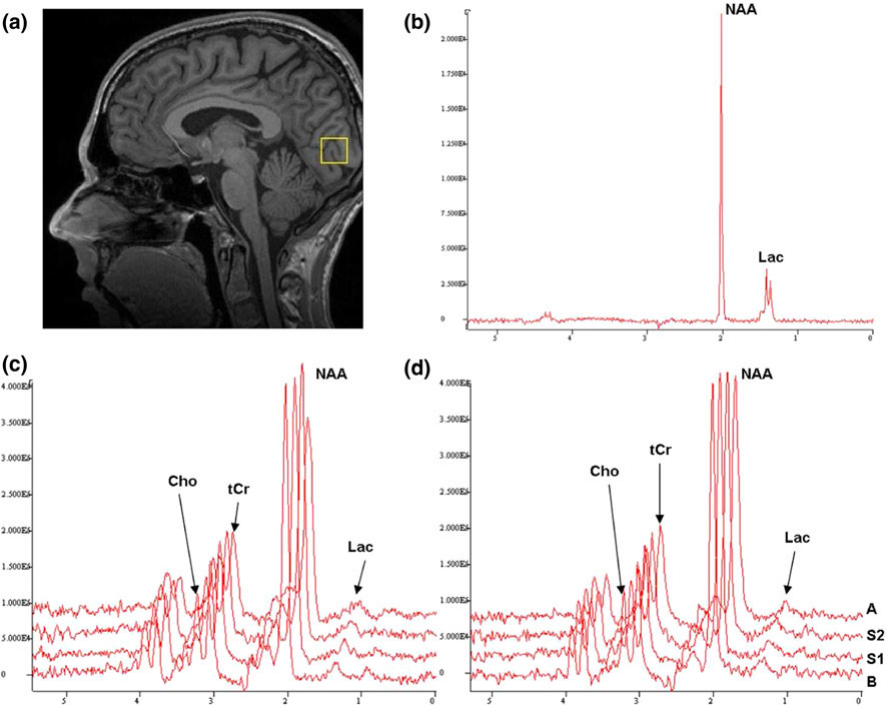
**a** Sagittal *T*_1_-weighted image with a 20-mm cubic VOI localized in the occipital visual cortex.** b** MR spectrum of a phantom containing NAA (2.01 ppm) and Lac (1.31 ppm).** c** Stack plot of spectra obtained before (B), during (stimulation 1, S1 and stimulation 2, S2) and following PS (after, A) in controls.** d** Stack plot of spectra obtained before (B), during (S1 and S2) and following PS (A) in MwoA patients. In vivo metabolites Lac (1.31 ppm), NAA (2.01 ppm), tCr (3.03 ppm) and Cho (3.19 ppm) are marked in the spectra

The volume of interest (VOI) was placed in the primary visual cortex (Brodmann area 17), centered on the calcarine fissure (Fig. 2a), localized on *T*_1_-weighted gradient-echo images in three orthogonal planes with a slice thickness of 1 mm, a repetition time (TR) of 1550 ms and an echo time (TE) of 2.37 ms. VOI size was $20 \times 20 \times 20\,\hbox{mm}^3.$ For each subject, 64 water-suppressed spectra (TE = 288 ms, TR = 2,000 ms, 8 averages), as well as 10 water-unsuppressed spectra at different TE (30, 50, 70, 90, 110, 150, 200, 300, 500, 1,000 ms) and a TR of 10,000 ms (1 average) were acquired. The raw data of each acquisition consisted of 1,024 complex-valued data points, at a sampling period of 0.833 ms. The corresponding bandwidth was 1200 Hz. The total duration of the examination was approximately 40 min.

PS was obtained by the projection of a black-and-white checkerboard, reversing at 8 Hz, and was viewed through MR-compatible goggles. Subjects were instructed to fixate a cross hair in the centre of the reversing checkerboard. The control condition consisted of the projection of a black screen. The experimental time course was: 6′24″ baseline phase (black screen) corresponding to 16 spectra; 12′48″ on phase (reversing checkerboard) corresponding to 32 spectra; and again 6′24″ baseline phase (black screen) corresponding to 16 spectra.

A phantom was used to evaluate the ^1^H-MRS protocol and contained an aqueous solution of 10 mM NAA and 1 mM Lac (Sigma Aldrich). Sodium azide ($\hbox{NaN}_3$) was added as an antimycotic agent. The phantom was made of plastic, was spherical and had a diameter of 10.4 cm. A spectrum obtained in the phantom is shown in Fig. 2b.

### Spectral analysis

Both water-suppressed and water-unsuppressed spectra were recorded in this study.

Water-unsuppressed spectra were processed without apodization and fitted by a single component using Hankel Lanczos Squares Singular Values Decomposition (HLSVD), a method based on the Lanczos algorithm and included in the jMRUI package [24], yielding an estimation of the water signal integral.

In the water-suppressed spectra, the residual water resonance was removed by HLSVD [25]. The signals of Lac, NAA, tCr and Cho were referenced at 1.31, 2.01, 3.03 and 3.19 ppm, respectively (Fig. 2c, d). The spectra were summed and averaged over 16 spectra, according to the corresponding condition. Following apodization (Lorentzian, 5 Hz) and phase correction, the water-suppressed spectra were fitted by the time-domain semi-parametric algorithm QUEST [26], software which is also included in the jMRUI package. QUEST is a non-linear least-squares algorithm that fits a time-domain model function, made up from a basis set of quantum-mechanically simulated whole-metabolite signals. These whole-metabolite signals were simulated with the software package NMR-SCOPE [27], which uses advanced prior knowledge and computes theoretical metabolite signals in terms of spin Hamiltonian parameters [28]. The PRESS sequence was simulated with NMR-SCOPE. Signal basis sets of NAA, tCr, Cho and Lac were created for a TE of 288 ms, so that the *J*-coupling modulations were the same as in vivo. We fitted all metabolites to reduce the effect of the spectral overlap. Each simulated metabolite spectrum was composed of multiple peaks from both the coupled and uncoupled protons.

### Quantification

Metabolite ratios of NAA/tCr, Cho/tCr, Lac/tCr and Lac/NAA were calculated using the signal integrals derived with QUEST.

Absolute in vivo concentrations, using the phantom replacement technique with NAA as a reference, were also calculated, using the following equation [29]:

1 $$ [C_i] = [C_r] {\frac{S_i V_r N_r c_{T_{1r}} c_{T_{2r}} T_i \rho_i c_{load}}{S_r V_i N_i c_{T_{1i}} c_{T_{2i}} T_r \rho_r c_{csf}}} $$

Subscripts *i* and *r* correspond with in vivo and the reference phantom, respectively, [*C*] is the metabolite concentration, the NAA metabolite concentration [*C*_*r*_] in the reference phantom was 10 mM, *S* is the signal integral (derived with QUEST), *V* is the volume of the voxel from which the signal is acquired, *N* is the number of protons that contribute to the spectral line (*N* = 3 for NAA, tCr and Lac, *N* = 9 for Cho), *c*_*T*1_ and *c*_*T*2_ are correction factors for the signal loss caused by longitudinal (*T*_1_) and transversal (*T*_2_) relaxation time, respectively, *T* is the absolute temperature ($T_i = 310.16\,\hbox{K}$ in the volunteer and $T_r = 294.16\,\hbox{K}$ in the reference phantom), ρ is the density of water, $\hbox{c}_{load}$ is a correction factor that accounts for different coil loading and *c*_*csf*_ is the correction factor for partial volume effects, i.e. the fraction of cerebrospinal fluid (CSF) compared to the fraction of water in the brain parenchyma in the VOI. The volume ratio $V_r/V_i$ cancels from the equation since *V* was the same in the reference phantom and in vivo, i.e. $20 \times 20 \times 20\,\hbox{mm}^3.$ For the calculation of the different correction factors, we refer to a recent publication on quantitative ^1^H-MRS in MwoA [11]. *T*_1_ and *T*_2_ relaxation times of Lac were derived from another study [30].

### Statistical analysis

Statistical analysis was performed using the SPSS software (SPSS 15.0 for Windows; Chicago, IL). Descriptive statistics were calculated for age, gender, signal-to-noise ratio (SNR), signal integral variations, metabolite ratios and absolute metabolite concentrations. To determine whether detectable changes in metabolite ratios and absolute metabolite concentrations occur in MwoA patients as compared to controls, before, during and after PS, a repeated measures ANOVA was performed. The metabolite ratios and concentrations were tested against the hypothesis of within-subject variations (i.e., condition) and between-subject variations (i.e., group). A least significant difference (LSD) test and a Scheffé test were used to evaluate possible group differences. Equality of variances was verified with the Levene’s test and normal distribution of data was evaluated by a Kolmogorov–Smirnov test. Results were considered to be significant for *P* < 0.05.

## Results

Spectra obtained during rest and stimulation conditions from both control subjects and MwoA patients are shown in Fig. 2c, d, respectively. Shimming resulted in linewidths around 8 Hz for water.

With the achieved SNR of the summed spectra at a TE of 288 ms, no other peaks than NAA, tCr, Cho and Lac were evident. On average the SNR was identical in the control and MwoA patient group (Table 1). SNR varied around 5% between the resting and stimulation phases ($\Updelta$SNR).

**Table 1:** SNR

	Controls	MwoA patients
SNR	2.4 ± 0.37	2.4 ± 0.35
$\Updelta$SNR (%)	5.2 ± 2.3	5.5 ± 2.5

In a phantom, signal integrals varied from <0.5% for NAA to 1–2% for Lac during the time course of the functional paradigm. Table 2 illustrates the total average in vivo signal integral variation per metabolite, as well as the average variation between the different conditions of the stimulation paradigm. The NAA signal integral has a coefficient of variation (CV) of approximately 3% during the course of the paradigm. For the tCr and the Cho signal, CV has somewhat higher values: around 7 and 11%, respectively. The highest CV, as expected, is found for the small Lac signal integral at around 30%. There are no differences between MwoA patients and control subjects for these abovementioned values (*P* > 0.05). When analyzing the signal integral variation between the three conditions, we observed considerable (for NAA and tCr) to very large (for Cho and Lac) standard deviations.

**Table 2:** Signal integral variation (%)

	Total^a^		Stimulation − Before^b^		After − Stimulation^c^		After − Before^d^	
	Controls	MwoA patients	Controls	MwoA patients	Controls	MwoA patients	Controls	MwoA patients
NAA	2.9 ± 1.7	3.0 ± 1.1	1.7 ± 3.3	1.3 ± 5.2	−2.5 ± 4.4	−1.4 ± 3.5	−0.8 ± 4.7	−0.3 ± 4.4
tCr	7.3 ± 4.4	7.7 ± 4.1	4.9 ± 14.4	4.5 ± 11.2	−4.9 ± 10.6	−0.5 ± 9.8	−1.4 ± 8.4	3.8 ± 14.1
Cho	11.9 ± 7.9	11.7 ± 7.1	8.1 ± 29.5	−0.3 ± 18.5	−2.4 ± 16.7	1.9 ± 19.5	3.1 ± 22.5	1.8 ± 28.7
Lac	33.4 ± 18.4	29.9 ± 17.2	16.9 ± 53.1	31.4 ± 74.5	7.9 ± 86.4	−5.8 ± 45.5	8.3 ± 55.9	−0.7 ± 42.8

^a^Total reflects the average signal integral variation of all conditions combined (before, during and after PS)

^b^Signal integral variation of the signals obtained before and during PS

^c^Signal integral variation of the signals obtained during and following PS

^d^Signal integral variation of the signals obtained before and following PS

The evolution of metabolite ratios and absolute concentrations during the stimulation paradigm are shown in Tables 3, 4, respectively. No significant differences in metabolite ratios, including Lac/tCr and Lac/NAA, and absolute metabolite concentrations, including [Lac], were observed between the different conditions in both the MwoA patient and the control group (*P* > 0.05). Also no significant differences were found between MwoA patients and controls, when looking at separate stimulation conditions (*P* > 0.05).

**Table 3:** Ratios

		Before^a^	Stimulation1	Stimulation2	After
NAA/tCr	Controls	2.34 ± 0.269	2.38 ± 0.289	2.26 ± 0.244	2.40 ± 0.239
	MwoA patients	2.46 ± 0.248	2.41 ± 0.233	2.42 ± 0.233	2.38 ± 0.256
Cho/tCr	Controls	0.221 ± 0.061	0.236 ± 0.059	0.214 ± 0.050	0.224 ± 0.036
	MwoA patients	0.222 ± 0.059	0.228 ± 0.047	0.221 ± 0.046	0.223 ± 0.045
Lac/tCr	Controls	0.242 ± 0.108	0.247 ± 0.118	0.270 ± 0.118	0.243 ± 0.117
	MwoA patients	0.267 ± 0.146	0.249 ± 0.099	0.288 ± 0.127	0.258 ± 0.122
Lac/NAA	Controls	0.103 ± 0.044	0.104 ± 0.048	0.120 ± 0.053	0.104 ± 0.054
	MwoA patients	0.112 ± 0.060	0.105 ± 0.043	0.126 ± 0.046	0.110 ± 0.050

^a^All conditions were averaged over 16 spectra

**Table 4:** Absolute concentrations (mM)

		Before^a^	Stimulation1	Stimulation2	After
$[\hbox{NAA}]$	Controls	14.92 ± 1.39	15.05 ± 1.39	14.75 ± 1.53	14.84 ± 1.70
	MwoA patients	15.22 ± 1.76	15.36 ± 1.88	15.30 ± 1.90	15.21 ± 1.80
$[\hbox{tCr}]$	Controls	12.68 ± 1.60	12.63 ± 1.69	13.14 ± 1.47	12.25 ± 1.47
	MwoA patients	12.43 ± 1.84	12.59 ± 1.84	12.54 ± 1.69	12.67 ± 1.59
$[\hbox{Cho}]$	Controls	0.88 ± 0.22	0.93 ± 0.17	0.89 ± 0.21	0.88 ± 0.18
	MwoA patients	0.92 ± 0.22	0.88 ± 0.18	0.89 ± 0.17	0.90 ± 0.18
$[\hbox{Lac}]$	Controls	0.51 ± 0.19	0.53 ± 0.23	0.60 ± 0.24	0.52 ± 0.24
	MwoA patients	0.55 ± 0.29	0.60 ± 0.24	0.62 ± 0.23	0.53 ± 0.26

^a^All conditions were averaged over 16 spectra

## Discussion

There were several good reasons to perform functional ^1^H-MRS in the visual cortex of MwoA patients.

The visual cortex is both easily stimulated and an interesting brain region because of its higher energy metabolism. The occipital lobe was found to have a significantly higher regional $\hbox{CMRO}_2$ as compared to other cortical regions [31], and, according to a PET study, the regional $\hbox{CMR}_{gl}$ was found to be the highest in occipital white matter and the visual cortex [32]. Migraine has been the subject of a large cohort of MRS studies (see reviews in [10, 11]). More specifically, Lac was found to be increased in the visual cortex at rest or following PS of MwA patients and migraine patients with a more complex phentotype (i.e., visual symptoms and one of the following symptoms: paraesthesia, paresia or dysphasia), respectively [12, 13, 14, 33]. A resting state ^31^P-MRS study in MwoA patients, performed by our group, demonstrated a decrease in high-energy phosphates (ATP and PCr) in the visual cortex, implying a reduction in aerobic metabolism [10]. Furthermore, no quantifiable Lac was observed in the same MwoA patient group, implying there is no significant switch to anaerobic glycolysis in the resting state [11]. The aim of this study was to elucidate whether Lac would increase in MwoA during and following PS.

In the present functional ^1^H-MRS study we did not observe any significant differences in signal integrals, ratios and absolute metabolite concentrations (including for Lac) between MwoA patients and controls, before PS. These findings are in contrast to observations in three studies covering MwA (including FHM and basilar-type migraine), which reported elevated basal Lac levels [12–14]. The authors of the abovementioned studies suggested that the accumulation of Lac was a marker for a disturbance in oxidative glycolysis, related to energy metabolism impairment, which is typical for mitochondrial diseases [34].

We also did not observe a significant increase of Lac during and following PS, both for MwoA patients and controls. In another comparative fMRS study between patients with MwA and migraine patients with both visual symptoms and paraesthesia, paresia and/or dysphasia (but not MwoA), a PS-induced Lac/NAA increase was reported only in the migraine patients with the more complex phenotype (i.e., visual and other symptoms) while no changes were observed in the MwA and control group [14]. It was postulated that the lack of stimulation-induced Lac increase in MwA and also in patients with mitochondriopathies might be partially attributed to a saturation of Lac transporter systems [35]. In the subgroup of MwA, with high resting-state [Lac] levels, it was hypothesized that the aura was limited to the visual cortex, whereas in the subgroup of migraine patients with both visual symptoms and paraesthesia, paresia and/or dysphasia, this was not the case. Experiments demonstrated that decreased pH, which would accompany increased extracellular Lac, is an inhibiting factor for cortical spreading depression, which is believed to be associated with the aura symptoms [36].

The lack of a significant increase in Lac in the healthy control population post-PS in our study is in contrast to some other early fMRS studies [19–21], in which PS-induced Lac increases of 60–150% were observed. However, several methodological issues have been raised concerning Lac visibility in MRS as well as Lac dynamics following PS, which are related to low Lac concentrations under different experimental conditions [37, 38]. This led to the need for optimization of the sensitivity and accuracy of fMRS methodology. Mangia et al. [39–41] reported about these issues in several advanced ^1^H-MRS studies and observed an increase in [Lac] of $0.1{-}0.2\,\mu\hbox{mol/g}$ in the first minute of PS, corresponding to an increase of only 20%. In contrast to most previous reports [19, 20, 21, 37, 38], these studies were performed at ultrahigh field strength (i.e., 7 T), resulting in larger chemical shift dispersions and higher SNR compared to lower field strenghts. Furthermore, in these studies data were acquired with ultrashort TE and a long TR, minimizing *T*_2_ and *T*_1_ relaxation effects, and, ultimately leading to very reproducible results with a high SNR. We acquired data using a long TE (i.e., 288 ms) as in several previous fMRS studies [20, 37, 38], resulting in low SNR spectra, large variability in Lac signal amplitudes, and consequently high standard deviations. Lac changes could not be identified at 3 T in the present study in both controls and MwoA patients, as the variations are lower than the sensitivity threshold of $0.1\,\mu\hbox{mol/g}$ (maximum 0.09 mM, see Table 4), determined in a functional ^1^H-MRS study in healthy volunteers at 7 T [39]. It should also be emphasized that the calculated absolute metabolite concentrations (i.e., around 0.5–0.6 mM) are in the range of what is assumed as normal for healthy brain (i.e., 0.2–1.0 mM); and Lac increases of up to 0.2 mM are unlikely to reflect a switch to anaerobic glycolysis, according to Mangia et al. [40].

In conclusion, we can state that in this study no obervable changes in Lac were found in MwoA patients, both at rest and following PS. This is in contrast to observations in MwA patients (and variants) as reported by others. This suggests that the observed Lac increase in previous MRS studies at 1.5 T in MwA is linked to aura, and therefore absent in our cohort of MwoA patients. It has to be mentioned that subtle metabolic changes (such as a small rise in Lac, smaller than $0.1\,\mu\hbox{mol/g}$) may not have been detected in healthy volunteers and MwoA patients, due to the used experimental conditions (3 T, sequence, long TE,$\ldots$). However, we performed a robust quantitative analysis in both a homogeneous MwoA patient group, and an age- and gender-matched control group. Our study argues against a significant switch to non-aerobic glucose metabolism during long-lasting PS of the visual cortex of MwoA patients.
